# Cytomorphometric Analysis of Oral Buccal Mucosa of Dental Colleges’ Students in Sulaimani City

**DOI:** 10.3390/diagnostics13020234

**Published:** 2023-01-08

**Authors:** Darya Khalid Mahmood, Ban Falih Ibraheem, Dena Nadhim Mohammad, Balkees Taha Garib, Marwa Abdul-Salam Hamied

**Affiliations:** Oral Pathology, Oral Diagnosis Department, College of Dentistry, University of Sulaimani, Sulaimani 46001, Iraq

**Keywords:** buccal mucosa, cytomorphometric analysis, hormonal influence, micronuclei

## Abstract

This study evaluates the cytomorphometric measures of cells obtained from the buccal mucosa of dental students to assess the fluctuation of the cellular characteristics among relatively normal subjects, with any potential correlations with demographic information, different habits, and hormonal disturbance. This prospective study included 100 dental students with no detectable oral alterations submitted to brush cytology. The smears were fixed with 95% ethyl alcohol and stained with hematoxylin and eosin stain. The stained section was observed under an image analyzer for cytomorphometric analysis. Cytopathological observations were recorded, including inflammation, microbial colonies, micronuclei, keratinization, overlapping, and hemorrhage. Chi-square tests were applied for non-parametric variables. One-way analysis of variance (ANOVA) was used to compare the cytometric parameters to habits and hormonal disturbances. A *p*-value < 0.05 was considered statistically significant. The results showed close proximity among subjects in the matter of cytomorphometric measures; no significant influence of sex, smoking, alcohol drinking habits, and menstruation was found on cytomorphometric diameters or cytopathological observation, and vice versa, while polycystic ovary syndrome impacted nuclear and nuclear-cytoplasmic ratio (*p* = 0.003, *p* = 0.02), respectively. Oral exfoliative cytology combined with cytomorphometric analysis for the studied normal individuals can be helpful in various investigations of oral and systemic diseases.

## 1. Introduction

Cytology is one of the best procedures applied for the initial microscopical examination of oral lesions [1]. It has proved high reliability and simplicity since its significance was first presented by Montgomery and Von Haam in 1951 [2]. Typically, epithelial cells are maintained firmly in place under normal circumstances. However, these cells might lose their cohesiveness in certain conditions, resulting in exfoliation [3].

Exfoliative cytology is the analysis of cells collected by normal shedding or artificial mechanical desquamation. It comprises various procedures, like brushing, imprinting, scraping, washing, and swabbing, all used to enrich the amount of sample obtained, thus facilitating a better collection and examination of smears [4]. Furthermore, brush exfoliative cytology has long been regarded as a simple, minimally invasive, and relatively painless approach and is well tolerated by patients. Therefore, it can detect changes in oral cells even in normal individuals and provides a promising option for the early detection of potentially malignant oral mucosal lesions [5]. Moreover, its importance has increased over the past years, synchronized with the emergence of new modalities, including immunocytochemistry, molecular analysis, advanced imaging techniques, and cytomorphometric analysis [6,7,8].

Cellular morphology reflects the biological behavior of the tissue and the genetic and molecular background of the cells themselves. Therefore, the slightest defect or alteration at the molecular level would launch a chain of reactions influencing the entire cell system, subsequently, its morphology [9]. This concept led to the application of cytomorphometry, a computer-assisted method for cell analysis. That is most widely used for assessing quantitative parameters of the cells, such as nuclear diameter/area (ND, NA), cytoplasmic diameter/area (CyD, CyA), and nuclear to cytoplasmic ratio (N/C ratio) [4]. These parameters have been shown to provide beneficial results in diagnosing diseases, some of which are systemic, such as anemia [10], diabetes mellitus [11], and hormonal changes [12].

Furthermore, evaluating the influence of external factors, like radiation [13], alcohol consumption [14], and smoking [9]. Meanwhile, other studies examined the associated cytomorphological changes in potentially malignant and malignant lesions [2,15]. However, little research has been concerned with evaluating the normal oral mucosa [16,17] as a baseline for comparison with pathological smears.

This study aims to do a cytomorphometric evaluation of the buccal mucosa of dental students to assess the fluctuation of the cellular characteristics among subjects. Furthermore, any potential correlations with demographic information, different habits, and hormonal disturbance are also reported. Such data can provide a database of potential cytological features in relatively normal individuals and the impact of having such statistics for comparison in future studies of different conditions and diseases.

## 2. Materials and Methods

### 2.1. Sample of the Study

Ethical approval was obtained from the Local Ethical and Scientific Committee in the College of Dentistry (proposal no:107/22, 11/4/2022) to conduct this prospective study at the laboratory of oral pathology/College of Dentistry.

Upon clinical examination, one hundred dental students with no detectable oral alterations were submitted to brush cytology. The demographic and clinical data for each student were registered in a case sheet. The recorded information included; sex, times of tooth brushing/mouthwash (if used) per day, habits (smoking, drinking), and history of hormonal changes for females, whether physiological (menstruation) or pathological (polycystic ovary syndrome PCOS). Verbal consent was obtained from students to participate in this study. The students were asked to rinse their mouths with water thoroughly. A disposable medium-hard nylon brush was sterilized in 0.2% of chlorhexidine gluconate mouthwash for 24 h. Under adequate illumination, the cytobrush was used with moderate pressure in one direction over the buccal mucosa numerous times until pinpoint bleeding was achieved, suggesting the penetration of the lamina propria. Thus allowing epithelial cells to be retrieved through the entire thickness of the epithelium. Cytology specimens were processed by a conventional method, in which the material from the brush was spread on the middle third of one clean, dried glass slide. The smears were then fixed immediately with 97% ethanol alcohol and stained with hematoxylin and eosin [1]. Two pathologists evaluated the slides under a light microscope for the following cytopathological findings: cellular overlapping, micronuclei, and keratinization. At the same time, the background was assessed for the presence or absence of hemorrhage, inflammatory cells, and microbial colonies. Dry slides or those with an insufficient sample of cells were excluded. As a result, seven cases were discarded, leaving a database of 93 participants.

### 2.2. Quantitative Cytomorphometric Evaluation

Different fields of each smear were captured using an AmScope auto-focus 1080 p digital c-mount camera at a magnification of ×10. The slide reading was performed stepwise, moving the slide from the left upper corner to the right and then down to avoid counting the same cell twice. Cells with defined outlines were measured, avoiding clumped or folded cells and those with distorted nuclei and cytoplasm. Cells from deep epithelial layers (intermediate or basal-parabasal) were considered adequate. The collected images of a maximum of 50 cells and a minimum of 30 cells were cytomorphometrically analyzed by using the image analysis software ImageJ version 1.44.

**Figure 1:**
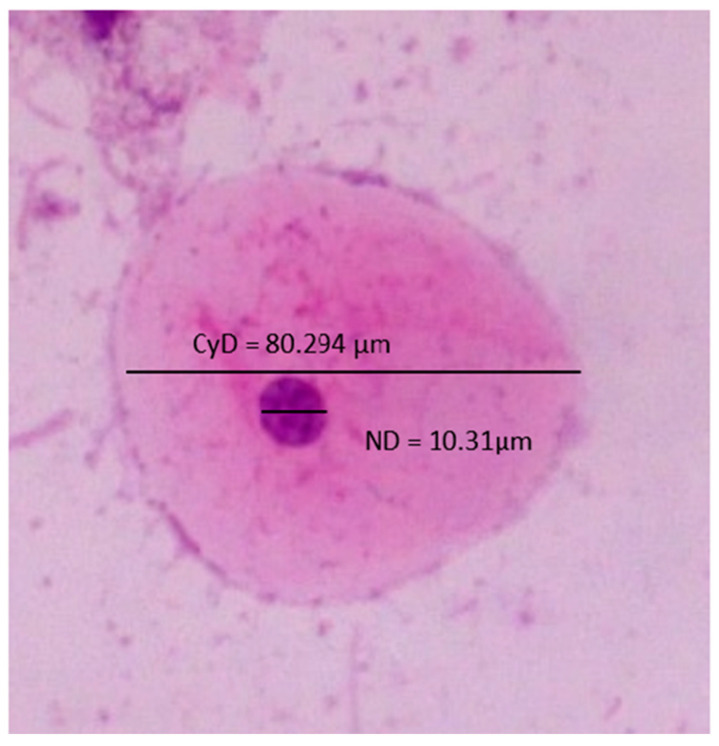
Cytological smear showing nuclear and cytoplasmic diameters.

Nuclear diameter (ND) and cytoplasmic diameter (CyD) were measured using a digitalized cursor with an interactive measurement tool by tracing two perpendicular lines (maximum and minimum diameter), which were measured by the software, and the mean values were recorded in micrometers (µm) (Figure 1). Meanwhile, the nuclear-to-cytoplasmic diameter (NCD) ratio was calculated using the formula: NCD = Nuclear diameter/cytoplasmic diameter [4]. Cytomorphometric evaluation was carried out in response to sex, habits, and female hormonal disturbances with the following criteria: the average nuclear diameter (ND), cytoplasmic diameter (CyD), and minimal and maximal diameters (D-min) (D-max), and NCD ratio.

### 2.3. Statistical Analysis

The results were statistically assessed using an IBM SPSS Statistics version 25.0 software for windows. The frequency, percentage, and Pearson Chi-square test were used for non-parametric variables. A *p*-value < 0.05 was considered statistically significant. One-way analysis of variance (ANOVA) was used to compare the parameters of nuclear diameter (ND), cytoplasmic diameter (CD), and the nuclear to cytoplasmic diameter (NCD) ratio among different groups.

## 3. Results

This study included 93 out of 100 fourth-stage dental students. Therefore, the participants shared the same age of 22 years old. In addition, the sample showed a female predominance of 58% (*n* = 54). And a male-to-female ratio of (1:1.4).

**Figure 2:**
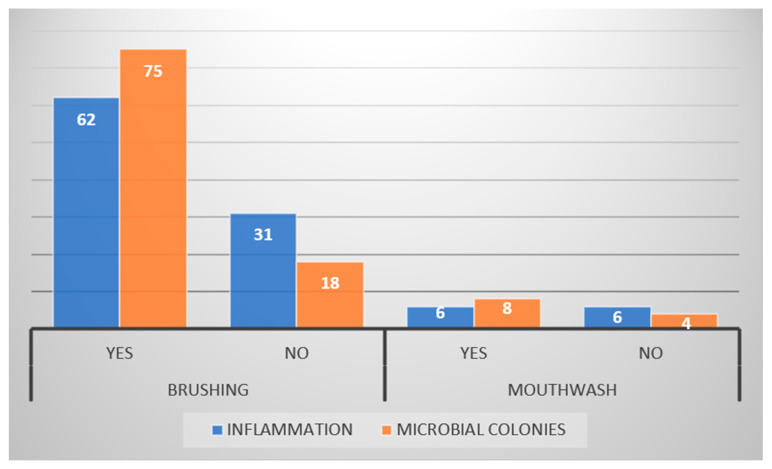
Oral health maintenance habits’ effect on cytopathological findings (no relation was found among the demonstrated variables). *p* > 0.05.

We found that 72% (*n* = 67) of participants followed brushing twice daily as an oral health maintenance routine, with only 12.9% (*n* = 12) using a mouthwash gargle. The smokers and alcohol drinkers were males, 12.9% and 5.4%, respectively. Regarding hormonal disturbance, 14.8% of female participants underwent menstruation at the time of sample collection, while only 7.4% were diagnosed with polycystic ovary syndrome. Interestingly habits and hormonal changes had significant roles in both sexes, as *p*-values were <0.05 (Table 1). This study assessed the frequency of cytopathological findings of inflammation and microbial colonies in terms of tooth brushing and mouthwash use. In which the majority experienced both inflammation and microbial colonies in their smears, among which the difference was not statistically significant (the reported *p*-value > 0.05) (Figure 2).

**Figure 3:**
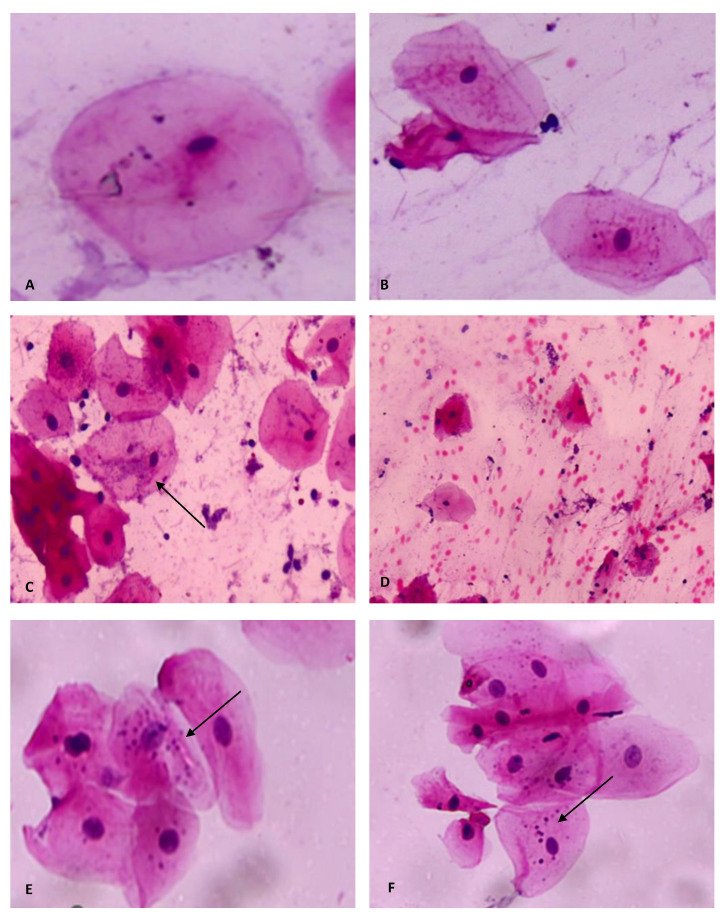
Cytological smears (X40), showing (**A**,**B**): Keratinization and cytoplasmic vacuolization. (**C**): Microbial colonies (arrow) inflammation and overlapping. (**D**): Hemorrhage and inflammation. (**E**,**F**): Micronuclei (arrows).

In terms of cytopathological findings, the entire sample revealed overlapping cells in all of the analyzed smears 100% (*n* = 93). Simultaneously, the majority established microbial colonies 80.6% (*n* = 75) and micronuclei 90.3% (*n* = 84). Moreover, 62.4% (*n* = 58) demonstrated signs of keratinization in the cytoplasm of cells, and 66.7% (*n* = 62) showed inflammation; meanwhile, only 10.7% (*n* = 10) showed hemorrhage. None of the reported findings were influenced by sex (Table 2 and Figure 3).

Most smokers established micronuclei 91.7%, followed by microbial colonies 83.3%, compared to non-smokers (8.3%, 16.7%). Meanwhile, only 50% showed inflammatory cells in the background of their smears. None of these findings were influenced by smoking, as *p*-values were > 0.05. Moreover, all smears of alcohol drinkers showed microbial colonies and micronuclei, and 80% exhibited keratinization. Additionally, the consumption of alcohol had no significant impact on those outcomes. Finally, the majority of females with hormonal disturbances, whether physiological or those with PCOS, showed predominate micronuclear findings, which were 87.5% and 100%, respectively. These hormonal changes did not impact the mentioned readings (Table 3).

Regarding cytomorphometric parameters, proximity was found among the different groups in terms of CyD, ND, and NCD ratios. However, in this study, PCOS-related hormonal alterations led to a larger ND, CyD, and NCD ratio compared to non-polycystic females and those with menstruation. In turn, no significant difference was observed between females with menstruation and normal ones. Additionally, sex, smoking, or drinking showed no statistical influence on the reported parameters except PCOS impact on ND and NCD ratio (*p* = 0.003, *p* = 0.02) (Table 4).

## 4. Discussion

Exfoliative cytology is a useful diagnostic modality that enables the early detection of abnormalities in the oral mucosa [15]. It is a simple, safe, and practical approach, particularly applicable for mass screening due to its high sensitivity and specificity [18]. Furthermore, the application of cytomorphometry offers remarkable development in the diagnosis and prognosis of serious diseases as it can detect cellular alteration, improving diagnosis accuracy and reproducibility [9]. Therefore, exfoliative cytology could be a beneficial adjunct to the clinical evaluation of lesions and influencing factors [19].

This study showed female predominance. However, since the sample was not collected haphazardly or in response to a particular disease or condition; instead, it was based on college admission within a specific age group. Therefore, it would be statistically unjustified to compare our data with other research on the matter of sex and age.

Most of our sample (72%) followed the international recommendations for a twice-daily frequency of tooth brushing; this could be attributed to the participant’s awareness of the significance of toothbrushing as a preventive measure and an indicator of oral hygiene [20]. Additionally, the procedure was sex-related since females expressed more interest in practicing the globally advised dental hygiene regimen, which agreed with the literature [21,22,23].

On the other hand, only 12.9% used mouthwash, in contrast to most of the participants, which was consistent with the findings of the study conducted by Macfarlane et al. [24,25]. This could be due to the controversy around mouthwash use and ongoing assessments of its general benefits and hazards.

This study revealed male predominance in terms of smoking and alcohol drinking habits, 12.9% and 5.4%, respectively. Other local and regional studies confirmed this issue in which most participants were males [3,26,27,28,29]. A finding that can be related to the conservative nature of the societies of middle eastern countries.

Regarding cytological observations of the obtained smears, 66.7% showed signs of inflammation; nevertheless, none of the reported habits nor hormonal changes influenced these findings. This disagreed with Queiroz et al. [17], no evidence of inflammation was seen in their sample of normal individuals. According to Seifi et al. [30], inflammation is considered one of the factors affecting nuclear and cytoplasmic size, as the author observed an increase in nuclear size and a decline in cytoplasmic size. Meanwhile, in our investigation, the inflammation had no noticeable effects on either the observational or cytomorphometric measures. This can be attributed to the type of the sample included, as Seifi conducted a study on smokers and waterpipe users with different oral site involvement, perhaps resulting in superimposed findings [30]. Ahmed et al. [31] mentioned that exposure to tobacco products could induce inflammatory events in the buccal mucosa, although, in his study, inflammation was noted in both smokers and control groups, with varying degrees of intensity. Additionally, Proia et al. [32] linked the presence of inflammation in response to bacterial infection; however, this cannot be supported by our findings as no direct correlation was discovered between the two observations. As a result of these contradicted findings, further investigation is required to uncover the underlying causes of inflammation and anticipate their potential consequences.

On the other hand, the majority of our sample (80.6%) showed bacterial colonies, which is higher than the findings of Abdelaziz and Osman [33] in their control group when compared to their groups of smokers and alcohol drinkers. Still, they ascribed their findings to these behaviors, which cannot be implicated in our case, as no significant association was found between these practices and bacterial existence. Moreover, this study showed keratinization in 62.4% of samples. This finding was unrelated to any of our sample’s habits or hormonal changes. This contradicted the findings of other studies [33,34] since no keratinization was observed in their control groups. Therefore they attributed the keratinization observation to smoking. Meanwhile, our results might be related to vigorous tooth brushing, the type of food intake, and perhaps accidental cheek biting.

The sample screening revealed 90.3% of micronuclei expression. As explained by the research, micronuclei originate from chromosome fragments or whole chromosomes, which stay behind at anaphase during nuclear division, and various genotoxic substances induce their formation [35]. Since 1983, many studies have used micronuclei detection as a short-term mutagenicity test. Since it is a simpler and much more rapid screening of chromosomal damage in cytological preparations [36], others used it as a reliable indicator for neoplastic progression [37,38].

Any increase in micronucleus count is a reflection of chromosomal alterations [39]. It has been reported that in normal healthy individuals, exposure to environmental pollutants such as drugs, chemicals, food, and free radical injuries, and lifestyle factors (smoking, alcohol consumption, diet, vitamin deficiencies) are related factors in producing the higher rates of micronuclei count in buccal mucosa and peripheral blood lymphocytes [40]. In contrast, no significant relation between micronuclei and drinking alcohol or smoking was discovered in our study. In the meantime, Al-Kasser [41] claims that Iraq’s environmental pollution has increased in recent years, which helps to explain our findings when combined with the aforementioned reasons. In light of the gravity of these observations, general awareness is required to spotlight the exact cause of such alarming findings.

Different alterations in the oral mucosa might result from smoking. It is therefore connected to various pathologies, ranging from fatal to reversible diseases [4]. According to published research, cytomorphometric analysis of smokers showed increased nuclear parameters and decreased cytoplasmic parameters compared to non-smokers [9,18,42]. In this study, despite the predominant micronuclei and keratinization in smokers and alcohol drinkers, no discernible difference was observed between smokers and non-smokers regarding morpho and cytometric findings. This could be due to the limited number of habit user participants and their youth, as these changes can be illustrated by continuous exposure to carcinogens over the years, resulting in reduced cytoplasmic maturation and significantly elevated nuclear activity [9,42]. On the other hand, Srinivasamurthy et al. [14] demonstrated that alcohol consumption had been associated with a considerable rise in nuclear, cytoplasmic, and nuclear to cytoplasmic parameters. However, this cannot be corroborated by our findings, as the number and the age of participants once more can be the main factor.

Research indicates that female hormones impact epithelial cell proliferation and development [3]. In general, ovarian hormones have a significant impact on females’ life. These hormones’ levels fluctuate throughout puberty, the menstrual cycle, pregnancy, and menopause [43]. It has been discovered that these hormones affect the oral cavity, as the onset of monthly menses evokes oral discomforts, including a burning sensation, bleeding with minor irritation, recurrent mouth ulcers, herpes labialis, and increased tooth mobility [43].

However, studies have not sufficiently proved a direct connection between alterations in the oral epithelium and hormonal changes during menstruation. The measuring of cytomorphometric characteristics in females based on their menstrual cycle was addressed by only three research. Balan et al. [44] discovered a significant difference in cellular and nuclear diameters during the different stages of the menstrual cycle of healthy young females. Meanwhile, in comparison with control groups, no significant changes were reported. At the same time, the other two studies [12,45] compared females with menstruation in different age groups. Further research emphasized that cytomorphometric parameters of the oral mucosa are undoubtedly affected by female hormones; hence, they excluded female members from their data to avoid superimposed results [30,46]. In the meantime, in this study, females with menstruation showed nonsignificant cytometric variation compared to non-menstruating females. Therefore, it is highly suggested that more studies be conducted on female hormones’ influence in different age groups on the oral mucosa to enrich the literature and provide sufficient data for comparison.

According to Deswal et al., 2020 systematic review, polycystic ovary syndrome (PCOS) is considered a common condition, occurring in 21.2% of reproductive-age females [47]. It has been linked to issues in the reproductive system and the metabolic system and an increased risk of cardiovascular diseases [47]. In the matter of PCOS influence on the oral cavity, the majority of investigations concentrated on the enhanced risk of developing the periodontal disease [48,49,50]. However, in this cytomorphometric study, in contrast to non-polycystic females and those with menstruation, PCOS -related hormonal alterations led to a larger ND, CyD, and NCD ratio, with an impact on the ND and NCD ratio (*p* = 0.003, *p* = 0.02). Since there is no research in the literature on the effects of PCOS on oral nuclear and cytoplasmic diameters, the precise cause of these observations cannot be quite explained. It can be recommended that future studies in this matter be conducted by physicians of both fields (cytology and gynecology) so that any conclusion would be more reliable.

## 5. Limitations and Conclusions

The lack of information regarding smoking and alcohol intake frequency was one of this study’s limitations. Furthermore, additional nuclear parameters with the number and size of micronuclei particles should be analyzed in a future study of the non-dental health groups in Sulaimani city to compare different lifestyle effects on cytomorphometric integrity. In conclusion, cytomorphometric analysis can be used in various fields of investigation due to its many potential benefits, including improved objectivity, enhanced sensitivity, and shorter turnaround times. This study provides a backbone of the record of data of normal individuals to be used as a baseline measurement for comparison with future cytometric studies. Minor nonsignificant cytomorphological alterations were related to habitual and hormonal alterations. Females with polycystic syndrome revealed a significant increase of nuclear and N/C ratio. Additional research with larger samples is required to support and validate these findings. The study sample expressed high micronuclei, indicating carcinogen doses from either local or ambient exposure. This was the most intriguing discovery. Exfoliated buccal cells can therefore be utilized as a marker for genotoxicity in the general population. In addition, this study can be used as a source of awareness in the population to reduce and avoid possible exposure to toxic factors.

## Figures and Tables

**Table 1:** Sex distribution concerning oral health maintenance, habits, and hormonal disturbances.

Variables		Male 39	Female 54	Total % 93	Pearson Chi-Square Test
Tooth brushing	Once	18 (19.4%)	8 (8.6%)	26 (28%)	*p* = 0.001
	Twice	21 (22.6%)	46 (49.4%)	67 (72%)	
Mouthwash	Yes	4 (4.3%)	8 (8.6%)	12 (12.9%)	*p* = 0.518
	No	35 (37.6%)	46 (49.5%)	81 (87.1%)	
Smoking	Yes	12 (12.9%)	0 (0%)	12 (12.9%)	*p* = 0.000
	No	27 (29%)	54 (58%)	81 (87%)	
Drinking alcohol	Yes	5 (5.4%)	0 (0%)	5 (5.4%)	*p* = 0.007
	No	34 (36.6%)	54 (58%)	88 (94.6%)	
Physical hormonal changes	Yes	0 (0%)	8 (14.8%)	8 (14.8%)	*p* = 0.000
	No	0 (0%)	46 (85.2%)	46 (85.2%)	
Polycystic ovary syndrome	Yes	0 (0%)	4 (7.4%)	4 (7.4%)	*p* = 0.000
	No	0 (0%)	50 (92.6%)	50 (92.6%)	

**Table 2:** Sex distribution concerning different cytopathological findings.

Variables		Male	Female	Total %	Pearson Chi-Square Test
Inflammation	Yes	25 (26.9%)	37 (39.8%)	62 (66.7%)	*p* = 0.500
	No	14 (15%)	17 (18.3%)	31 (33.3%)	
Microbial colonies	Yes	34 (36.6%)	41 (44%)	75 (80.6%)	*p* = 0.111
	No	5 (5.4%)	13 (14%)	18 (19.4%)	
Keratinization	Yes	27 (29%)	31 (33.4%)	58 (62.4%)	*p* = 0.245
	No	12 (12.9%)	23 (24.7%)	35 (37.6%)	
Micronuclei	Yes	37 (39.8%)	47 (50.5%)	84 (90.3%)	*p* = 0.207
	No	2 (2.2%)	7 (7.5%)	9 (9.7%)	
Overlapping	Yes	39 (41.9%)	54 (58.1%)	93 (100%)	
Hemorrhage	Yes	4 (4.3%)	6 (6.4%)	10 (10.7%)	*p* = 0.896
	No	35 (37.6%)	48 (51.7%)	83 (89.3%)	

**Table 3:** The distribution of habits and hormonal changes in response to various cytopathological findings.

Variables		Inflammation	Microbial Colonies	Micronuclei	Keratinization	Pearson Chi-Square Test
Smoking (12, 12.9%)	Yes	6 (50%)	10 (83.3%)	11 (91.7%)	9 (75%)	*p* = 0.260 *p* = 0.951 *p* = 0.866 *p* = 0.333
	No	6 (50%)	2 (16.7%)	1 (8.3%)	3 (25%)	
Alcohol drinking (5, 5.4%)	Yes	2 (40%)	5 (100%)	5 (100%)	4 (80%)	*p* = 0.430 *p* = 0.258 *p* = 0.452 *p* = 0.403
	No	3 (60%)	0	0	1 (20%)	
Physical hormonal changes (8, 14.8%)	Yes	4 (50%)	6 (75%)	7 (87.5%)	5 (62.5%)	*p* = 0.644 *p* = 0.304 *p* = 0.451 *p* = 0.484
	No	4 (50%)	2 (25%)	1 (12.5%)	3 (37.5%)	
Polycystic ovary syndrome (4, 7.4%)	Yes	2 (50%)	3 (75%)	4 (100%)	2 (50%)	*p* = 0.452 *p* = 0.240 *p* = 0.298 *p* = 0.484
	No	2 (50%)	1 (25%)	0	2 (50%)	

**Table 4:** The relation of sex, habits, and hormonal changes to cytoplasmic, nuclear diameters, and nuclear/cytoplasmic ratio.

Variables	Gender		Smoking		Drinking Alcohol		Physical Hormonal Changes		Polycystic Ovary Syndrome	
	Male	Female	Yes	No	Yes	No	Yes	No	Yes	No
CYD	66.341	65.971	66.399	66.086	64.223	66.234	65.441	66.064	66.326	65.943
MIN/MAX	56.20/77.75	48.20/81.26	58.02/76.29	48.20/81.26	58.02/76.23	48.20/81.26	53.27/78.56	48.20/81.26	56.65/78.01	48.20/81.26
ND	8.149	8.201	8.013	8.203	8.178	8.179	8.366	8.172	9.461	8.099
MIN/MAX	6.80/9.89	6.22/10.24	7.53/8.87	6.22/10.24	7.53/8.87	6.22/10.24	7.16/10.24	6.22/10.04	8.70/10.24	6.22/9.93
NCR	12.351	12.543	12.179	12.506	12.828	12.443	12.875	12.485	14.468	12.389
ANOVA	ND *p* = *0*.755 CyD *p* = *0*.806 N: C *p* = *0*.549		ND *p* = *0*.435 CyD *p* = *0*.887 N: C *p* = *0*.486		ND *p* = *0*.998 CyD *p* = *0*.540 N: C *p* = *0*.580		ND *p* = *0*.775 CyD *p* = *0*.946 N: C *p* = *0*.668		ND *p* = *0*.003 CyD *p* = *0*.965 N: C *p* = *0*.023	

## Data Availability

All the data are included in the manuscript.
